# Nanomaterials in Wound Healing and Infection Control

**DOI:** 10.3390/antibiotics10050473

**Published:** 2021-04-21

**Authors:** Ali Pormohammad, Nadia K. Monych, Sougata Ghosh, Diana L. Turner, Raymond J. Turner

**Affiliations:** 1 Department of Biological Sciences, Faculty of Science, University of Calgary, 2500 University Dr. N.W., Calgary, AB T2N 1N4, Canada; ali.pormohammad@ucalgary.ca (A.P.); nkmonych@ucalgary.ca (N.K.M.); 2 Department of Microbiology, School of Science, RK University, Rajkot 360020, India; ghoshsibb@gmail.com; 3 Department of Family Medicine, Cumming School of Medicine, University of Calgary, Calgary, AB T2N 1N4, Canada; dturner23@me.com

**Keywords:** nanoparticles, antimicrobial nanomaterials, infection control, wound healing, wound management, antimicrobial activity, infectious microbes

## Abstract

Wounds continue to be a serious medical concern due to their increasing incidence from injuries, surgery, burns and chronic diseases such as diabetes. Delays in the healing process are influenced by infectious microbes, especially when they are in the biofilm form, which leads to a persistent infection. Biofilms are well known for their increased antibiotic resistance. Therefore, the development of novel wound dressing drug formulations and materials with combined antibacterial, antibiofilm and wound healing properties are required. Nanomaterials (NM) have unique properties due to their size and very large surface area that leads to a wide range of applications. Several NMs have antimicrobial activity combined with wound regeneration features thus give them promising applicability to a variety of wound types. The idea of NM-based antibiotics has been around for a decade at least and there are many recent reviews of the use of nanomaterials as antimicrobials. However, far less attention has been given to exploring if these NMs actually improve wound healing outcomes. In this review, we present an overview of different types of nanomaterials explored specifically for wound healing properties combined with infection control.

## 1. Introduction

The skin is the largest organ of the body with many functions ranging from immunologic and sensorial to acting as a critical barrier for protection and moisture control [1,2]. The prevalence of different wounds continues to be a serious concern due to the increasing incidence of injuries, surgery, burns and chronic diseases such as diabetes [1,2]. Based on the latest report from the World Health Organization (WHO), more than 300,000 deaths occur annually due to burn wounds alone [3]. After skin damage, the immunologic and protective mechanisms of the skin are lost; consequently, microorganisms can easily infect the wounded site and may lead to chronic infection or septicemia if the organisms infiltrate the body. Moreover, infecting microorganisms can cause delays in the healing process by producing a biofilm, leading to difficulties in treatment due to antibiotic resistance and resulting in life-threatening complications [3,4]. Simultaneously, the presence of multidrug-resistant (MDR) organisms has presented a significant clinical challenge due to reduced antimicrobial treatment options [5]. The longer a wound healing process takes, there is a higher clinical risk for a serious adverse event, from amputations, organ loss and death.

According to predictions from the Centers for Disease Control and Prevention (CDC), the mortality rate from antimicrobial-resistant (AMR) and multidrug-resistant (MDR) infections will be higher than all types of cancer combined. It is estimated that AMR could lead to >10 million deaths a year by 2050 [6]. Treating wound infections with traditional antibacterial drugs is a growing concern with many clinical challenges [7]. Therefore, the development of novel wound dressing materials that do not rely on conventional antibiotics is critical.

The application of nanomaterials (NM), particularly nanoparticles (NPs), in wound healing and infection prevention has increased remarkably over the past 2 decades [8,9,10]. Recently, combining NPs into scaffolds for wound healing represents the targeted approach of advanced dressings that has gained significant attraction [7,10]. There are now a variety of antimicrobial NMs, from polymers such as lipids as liposomes [11], cellulose [12] or mesoporous silica [13] that are used as carriers of the antimicrobial agent. Another group of antimicrobial NM are metal/metalloid-based nanoparticles (MNPs), where several studies have reported to aid in wound healing [3,7,14]. This wound healing may or may not be directly related to their excellent antimicrobial potency [3,15,16]. Although the idea of nano-antibiotics (also as nanobots) has been around for a decade at least [17] and many recent reviews of the use of nanomaterials as antimicrobials [17,18,19,20,21], or antiseptics [22] exist, less attention has been given to explore if they actually improve wound healing outcomes.

Although there are now countless reviews exploring the antimicrobial efficacy of various nanomaterials, only a few reviews have been published on the topic of nanomaterials specifically used to improve wound healing [2,3]. Recently, an excellent recent review presented by Stoica et al. [10] overviewed NM in wound dressings. Here, we aim to complement these other works and present a review of studies that evaluated not only the antimicrobial efficacy of the NM, but also measured wound healing processes. Such an overview is important to evaluate NM true potential if we are to move them from academic curiosities to be regularly employed in the clinical setting.

## 2. Wound Processes

Below follows a brief overview of wound types and the healing process in order to provide context to how nanomaterials could influence healing.

### 2.1. Wound Categories

Wounds are defined as damage to the integrity of biological and organic tissue, including skin and mucous membranes [23]. Various types of trauma are possible such as burns, incision, laceration, contusion, abrasion and combinations and appropriate cleaning and wound dressing for each type is critical to limit the spread of infection and further injury [2,23,24]. Different methods are used for wound classification based on cleanliness, wound condition and healing time. The CDC classifies wounds into four categories based on wound cleanliness and level of infection; (1) Clean wounds: closed, no infection and inflammation. (2) Clean-contaminated: lack of unusual contamination. (3) Contaminated: fresh, open and lack of purulent inflammation. (4) Dirty-infected: most common microorganisms are present in these kinds of wounds and typically it results from improperly cared for traumatic wounds [25,26]. Further classification separates wounds into either (1) Acute wounds: short term, more common, often caused by irradiation, heat, electrical shock, or mechanical damage, or (2) Chronic wounds: long term, often a complication of chronic illnesses such as diabetes [2,27].

### 2.2. Wound Healing Process

Wound healing is complicated and can progress forward or backwards depending on different factors such as patient external and internal conditions (3). Wound healing is divided into four overlapping phases:

(1) Hemostasis: this stage happens immediately to form a blood clot to reduce bleeding [3,28].

(2) Inflammation: During this phase, bleeding is controlled and microorganisms and damaged cells are removed from the damaged region. Platelets are activated by thrombin and release several growth factors, signals to attract white blood cells, nutrients and more growth factors that accelerate wound healing and protect skin from infection [3,28].

(3) Proliferation: During this phase, the wound is renovated, starting with proangiogenic factors released by inflammatory cells and platelets. Then, angiogenesis occurs, fibroblasts proliferate and elastin is produced. Fibroblasts differentiate into myofibroblasts causing the contraction of the wound area by gripping the wound edges [3,28,29].

(4) Maturation: the wound is now fully closing with the help of collagen fibers. The cells and debris used to repair the wound, which are no longer needed are removed by programmed cell death or apoptosis. The skin of the injured area becomes stronger by collagen cross-linking [3,28].

### 2.3. Available Therapeutic Options in Wound Healing

Currently, available therapeutic options include biological-based approaches such as immune-based antimicrobial molecules (e.g., polypeptide like defensins), therapeutic microorganisms (e.g., bacteriophages and probiotics), stem cell therapy and skin tissue engineering. Traditional and non-traditional non-biological approaches include reactive oxygen species and nitric oxide generators, various topical antiseptics and antibiotics, negative pressure, ultrasound therapy, as well as physical wound debridement, various surgical approaches and skin substitutes [3,30,31].

## 3. Application of Different Nanomaterials in Wound Healing

Treatment, skin regeneration and prevention of infection are complex processes in the wound healing process. Both local and systemic treatments are used in wound healing. However, local treatment could be a preferable method to prevent side effects, increase efficacy and overcome antibiotic resistance [32]. Studies have shown that some pharmaceutical NPs have impressive potency in accelerating skin regeneration, as well as infection prevention and antimicrobial activities against MDR isolates [3,31]. Developing, commercializing and clinical application of these NM compounds could lead to remarkable improvements in wound treatment [3,31].

The application of local antimicrobial therapy increases the effective concentration of antibiotics and consequently leads to effective eradication of wound infection. Therefore, for the prevention of antimicrobial side effects and drug-resistance, ideally, antibiotics should be limited to site applications rather than systemic and long-term treatments [33]. In wound injuries, the skin loses its barrier function; thus, any topically applied drugs such as NPs, could be absorbed systemically. Thus, eukaryotic cell cytotoxicity and possible systemic side effects of NPs as nano-antimicrobials or nano-carriers should be considered in their pharmacokinetics and pharmacodynamics. Additionally, particular to topical applications, consideration towards the NPs half-time in the wound site is required, which is also influenced by the site cleaning rituals [3,31].

Below follow the different types of nanomaterials for wound treatment. Selected case studies are given for each type as examples of proof in concept; however, we do not provide an exhaustive listing of all studies with a given nanomaterial.

### 3.1. Core-Shell Nanoparticles and Surface Nanoengineering

Nanomaterials are synthesized in a wide variety of sizes, shapes and structures. The most commonly used are between 10–100 nm in size and roughly spherical [34]. Nanomaterial can be made from a variety of organic chemicals and metals. Most of the NPs typically contain two components, the core molecules or atoms and a surface “cap” or shell that influences the stability of the NPs as well as their function [35]. Various NM features such as the nanomaterial type(s), formulation and NP size, along with the degree of wound injury, contribute to the NPs efficacy in interacting with both the infectious organism and tissue cells in the injured area [36].

Some NPs are the active agents intrinsically, others are carriers of the antimicrobial or inflammation control drug. NPs that act as a carrier are materials performing a function as a vehicle to bring antimicrobials/drugs to the site of action in a more effective manner versus the application of the drug alone [37]. These may also be referred to as nanobots. These carrier NPs may be organic (e.g., surfactant/lipid), carbohydrate-based (e.g., cellulose) or inorganic (e.g., silicate or metal-based) [37,38,39,40]. The carrier NP may encapsulate the antimicrobial/drug as in liposome-based nanomaterials (Section 3.3), or the NP is the carrier via the antimicrobials being part of the shell [41]. This is the idea of core-shell nanostructures in drug delivery.

There are a few concerns that should be considered in the application of NPs as a carrier. The carrier core NP may be toxic and thus the shell needs to shield the NP from the biological system in order for the final combination to be non-toxic, non-allergenic, non-immunogenic, biodegradable (yet biocompatible on the time frame of wound healing application) and should release the NPs/antimicrobial at the injured site in a controlled fashion [37,41].

Related to core-shell nanomaterials, is that of surface nanoengineering aimed at preventing biofilm formation which occurs in wounds as well as infections on medical indwelling devices. The idea of surface nanoengineering is to produce a coating material that either passively prevents bacterial growth or active strategies that have antimicrobials attached to the polymer permanently (non-release based) or releases them (either with or without bacterial stimulation). Two recent reviews overview the chemistries around such polymer coatings [42,43]. Similar chemistries can and are, be applied to the shells of the inert core nanomaterials.

### 3.2. Carbon-Based Nanomaterials

Carbon based materials continue to gain interest in a variety of fields as they tend to have diverse physicochemical properties. The term carbon dot (CD) is a general term used for various nanoscale carbon materials such a graphene, polymers, rods, sheets and fullerenes. There are a variety of approaches to synthesize CD and can be functionalized or even dopped with metal-based antimicrobials (Section 4) [44].

#### 3.2.1. Carbon Quantum Dots

Here, we specifically consider the newest member of the carbon-based nanomaterials that are referred to as carbon quantum dots (CQD) which are considered zero-dimensional carbon material. These have been found to have antibacterial and antibiofilm efficacy [44]. A recent study had CQDs in a chitosan dextran hydrogel, which turned out to be an excellent self-healing matrix [45]. This combination reduced the inflammation response and promoted wound repair as displayed by the healing rate, granulation tissue thickness and collagen disposition. This is a new and exciting area and we expect we will see more in this area.

#### 3.2.2. Carbon Nanotubes

Carbon nanotubes (CNTs) are one of the most preferred nanocarriers, owing to their large specific surface area that can be exploited for efficient drug adsorption, either in the tube or bonded on the surface. Rational modification of CNTs can enhance drug encapsulation efficiency and stability while reducing drug release rate and cytotoxicity [46,47]. Chen et al. [48] reported isoniazid (INH)-loaded chitosan (CS)/carbon nanotubes microspheres with the ability to promote secondary wound healing in bone tuberculosis of guinea pigs initially infected subcutaneously with Mycobacterium tuberculosis [48]. This is an important finding, as secondary wound healing of chronic wounds is difficult to deal with. Reduction in tuberculosis ulcer by INH/CS/CNTs nanoparticles was 94.6% and 89.8% more compared to treatment with bare INH and INH/CNTs, respectively. These nanoparticles controlled the immunological responses by decreasing CD3+ and CD4+ T cell numbers.

In another study with CC-72 mice cell line, enhanced wound healing activity was observed when single-wall (SW) or multi-wall (MW) carbon nanotubes were complexed with chitosan, producing conjugates showing an interweaving network of CNTs with the chitosan polymer [49]. Results revealed 80% of the wounds treated with 1% C-SW-CNTs or 5% CS-MW-CNTs and 60% of those treated with 5% CS-SW-CNTs had more fibrosis compared to the internal control wounds. However, only 40% of the wounds treated with chitosan or 1% CS-MW-CNTs exhibited more fibrosis. Enhancement of wound healing by both types of complexes was attributed to the induction of improved re-epithelialization.

Santos et al. reported superior wound healing properties of nanocomposites polyvinyl alcohol/functionalized-MW-CNTs conjugated with glucose oxidase [50]. The bionanocomposite with this enzyme showed bioactivity toward injecting glucose with simultaneous antimicrobial behaviour against bacterial pathogens due to the generation of hydrogen peroxide from the oxidase.

It is important to note that CNTs exhibit better wound healing properties than other non-metallic nanostructures owing to its property to promote cell migration when incorporated in hydrogels. Biomedical applications of hydrogels made from various polymers are well accepted because of their unique properties that include biocompatibility, biodegradation, mechanical stiffness, hydrophilicity and porosity [51]. An interesting study conducted by Ravanbakhsh et al. [52] incorporated multiwalled carboxylic (COOH) functionalized CNTs with a diameter of 50–80 nm in fibrous composite hydrogels [52]. The CNT-loaded glycol-chitosan (GC) hydrogels recruit the fibroblasts better than the pure GC hydrogel. The hydrogel containing 250 µg/mL of CNT/GC (CNT250 group) exhibited the highest rate of cell migration [52]. MW-CNTs may serve as focal adhesion sites to enhance cell adhesion for anchorage-dependent cells such as fibroblasts that in turn facilitates the cell migration rate. Moreover, the larger diameter of MW-CNTs is advantageous over SW-CNTs as it approximates the physical characteristics of fibrous proteins, such as collagen and elastin, in the extracellular matrix (ECM). These studies suggest that MW-CNTs delivered in hydrogels have incredible potential as a powerful wound healing nanomedicine.

#### 3.2.3. Graphene

Graphene is a carbon matrix consisting of a single layer and two-dimensional lattice that gives a large surface area, high thermal and electrical conductivity, excellent mechanical strength and superior biocompatibility [53]. Graphene can be incorporated in hydrogels to enhance the mechanical properties and also porosity which in turn enhances water absorption capacity. Further, graphene-based hydrogels may have excellent biocompatibility and facilitate cellular adhesion and growth. Wound healing can be expedited as graphene hydrogels can maintain the moist environment. Thus, graphene is considered for fabricating cellular scaffolds, drug carriers, biosensors, stimuli-responsive actuators, supercapacitors and catalytic bulk materials. Recently, graphene-based composites have been explored for wound healing properties [54].

Fan et al. reported an antimicrobial silver–graphene nanocomposite with wound healing properties in rat models [55]. Graphene oxide (GO) colloid solution was prepared from graphite which was further supplemented with various concentrations of silver (Ag) in order to achieve composites with different Ag/graphene weight ratio (0.5, 1 and 5), denoted as Ag0.5G1, Ag1G1 and Ag5G1, respectively. The incorporation of silver nanoparticles (AgNPs) in the hydrogels prepared in situ, enhanced the antimicrobial activity of both Ag1G1 and Ag5G1 hydrogels significantly against *E. coli* and *S. aureus*. In vivo studies also showed significant wound healing after being treated by Ag1G1 and Ag5G1 hydrogels, particularly withAg5G1 exhibiting a wound healing ratio of 98%, compared to Ag1G1 hydrogel treated and control groups only showing 85% and 53% healing, respectively. The high wound-healing effect of Ag5G1 hydrogel was speculated to be attributed to the synergistic effects of both GO and AgNPs.

Fu et al. demonstrated, in the streptozotocin induced diabetic mouse model, that reduced graphene oxide (RGO) nanoparticles can be effectively incorporated into an acellular dermal matrix (ADM) that acted as an efficient natural scaffold for transplanting mesenchymal stem cells (MSCs) in diabetic wound healing [56]. ADM scaffolds, crosslinked with 1-ethyl-3-(3-(dimethylamino)propyl)carbodiimide hydrochloride (EDC) and N-hydroxysuccinimide (NHS), were used with GO or RGO to self-assemble on these scaffolds. The resulting self-assembled 3D multi pore hybrid scaffolds showed the diameters of collagen fibrils in a range from 92.22 ± 10.89 nm, 106.11 ± 9.71 nm and 107.55 ± 11.71 nm for ADM, ADM-GO and ADM-RGO, respectively. On the 14th day the control, ADM-MSC, ADM-GO-MSC and ADM-RGO-MSC groups showed 82.60% ± 1.69%, 90.26% ± 1.07% and 93.61% ± 2.76% wound closure, respectively. Thicker granulation tissue was observed to be formed on postoperative day 14 in the wounds treated with the ADM-RGO-MSC. The same group also exhibited thicker epidermal tissue at 14 days that decreased dramatically at 28 days. ADM-RGO-MSC treated groups also showed more collagen regeneration in wound sites confirming the fact that these novel hybrid scaffolds may be a promising biomaterial for the treatment of chronic diabetic wounds.

In another study, porcine-derived gelatin was reacted with methyl acrylic anhydride (MA) to yield a white porous foam-like GelMA prepolymer. This GelMA based hydrogel was loaded with different concentrations of RGO and cross-linked using UV light. This resulted in hydrogels of large porous structure (pores of 50 μm and RGO size of 30–40 nm) [57]. The wound healing efficacy of the hydrogel was evaluated by investigating the potential to enhance the migration of 3T3 fibroblasts, EA.hy926 endothelial cells and Hacat keratinocytes. Significant wound healing properties was seen with fibroblasts on treatment with 0.001% *w/w* and 0.002% *w/w* RGO loaded GelMA hydrogel. Migration of endothelial cells, keratinocytes and 3T3 fibroblast cells were observed on treatment with 0.002% *w/w* RGO loaded GelMA hydrogel. Migration (up to 85%) in 3T3 fibroblast cells indicated the excellent wound healing property of the RGO loaded GelMA hydrogel.

Tang et al. [58] reported, in the Sprague Dawley (SD) rat model, an excellent wound healing potential of polydopamine (PDA)-reduced graphene oxide (pGO)-incorporated chitosan (CS) and silk fibroin (SF) (pGO-CS/SF) scaffold with good mechanical, electroactive and antioxidative properties. It is important to note that the pGO acted as nano reinforcement and thereby enhanced the mechanical properties of the scaffold. Moreover, uniformly distributed pGO in the scaffolds facilitated the transmission of electrical signals. Another advantage of incorporating pGO in the scaffolds was scavenging reactive oxygen species (ROS), thus eliminating excessive oxidative stress. Wound closure areas in the pGO-CS/SF group were greater compared to the CS/SF group as observed on days 7 and 14. Further, the wound area closure rate in the pGO-CS/SF group and the CS/SF group reached 85 and 65%, respectively, which was ~25% greater than the control group at 14 days. Hence, the pGO-CS/SF scaffold can be used as a promising wound dressing material for promoting fast recovery through improved wound regeneration effect.

### 3.3. Liposomes

Liposomes (LPs) are considered as excellent delivery systems for hydrophilic molecules such as charged and small molecules like peptides and non-Lipinski [59] macromolecules [11]. The amphipathic nature of LPs is attributed to the phospholipid building blocks. On dispersing in aqueous solutions, the amphipathic lipids spontaneously form a lipid bilayer with an aqueous core. Lipid type and extrusion through membranes of various pour size will dictate the final size of the liposome. Further, these LPs can be functionalized with biopolymers in order to enhance stability and targeting properties.

Mengoni et al. reported chitosan-coated liposomal formulations for delivery of substance P (SP), which is a neuropeptide of 11 amino acids to facilitate mucosal wound healing [60]. Liposomes were prepared using lecithin and cholesterol and loaded with SP which was then resuspended in water; chitosan coated preparations were then made. The uncoated liposomes (UN-LP) had a mean diameter of 151 ± 27 nm, while the coated systems (CH-LP) had a diameter of 243 ± 24 nm. In vitro wound healing potential of the SP loaded chitosan functionalized liposomes was evaluated against HaCaTkeratinocyte cells. Tracking of cell migration revealed that SP encapsulated in chitosan-coated liposomes resulted in wound closures of 85.5 ± 12%, which was significantly higher compared to the untreated cells (45 ± 2.36%).

Recently, Ternullo et al. demonstrated wound healing properties of the natural plant extract curcumin. This was delivered in deformable liposomes (DL)-in-chitosan-hydrogel [61]. Both DLs and chitosan hydrogel can ensure sustained release and better penetration of the drug. Combination of both can result in prolonged retention time at the targeted skin site, improved localized wound therapy and enhancement in drug efficacy.

Danggui Buxue decoction (DBD), a popular Chinese traditional medicine, is composed of Danggui (Angelica sinensis, AS) and Huangqi (Astragali radix, AR) at a ratio of 1:5 (g/g) and is known for “invigorating Qi and promoting Xue,” suggesting that it has potential for wound healing. Cui et al. showed DBD–loaded liposomes in thermosensitive gel (DBLTG) had superior wound healing [62]. DBLTG was applied on dorsal full-thickness excisional wounds in adult male Sprague-Dawley (SD) rats. Treatment with DBLTG showed faster wound closure on day 7 and day 14 compared to untreated control groups. Wound contraction in the rats on topical application of DBLTG was found to be 32.03 ± 3.81%, 64.22 ± 2.31% and 92.98 ± 3.64% on day 3, day 7 and day 14 after surgery, respectively, which was significantly higher than the control group. Similarly, CD34 expression in granulation tissues increased on treatment with DBLTG. The enhanced wound healing efficacy of these novel liposomes also involved the vascular endothelial growth factor (VEGF)/phosphatidylinositol 3-kinase (PI3K)/Akt and transforming growth factor-beta (TGF-β)/Smads signalling pathways. Moreover, significant increases in the density of blood vessels, cells proliferation and expression of type I and type III collagen were observed in DBLTG treated wounds.

### 3.4. Metal(Loid)-Based Nanoparticles

Several metals have been used for centuries as antimicrobial agents and have been shown to have good efficacy not only against planktonic cells but also biofilms [63]. Metal and metalloid atom-based nanoparticles (MNPs) are also used in many areas of medicine for diagnostics, medical imaging, drug delivery and antimicrobial agents [64,65]. Because of the high surface area/volume ratio and surface-active properties, MNPs are often more effective than their salt and metal alloy counterparts in their antimicrobial properties. Consequently, several MNPs have been explored for their antimicrobial efficacy to control not only planktonic cells, but also those growing as a biofilm. MNPs have a feature to eradicate existing biofilms and inhibit biofilm formation [66,67,68,69,70].

Wound dressings have a wide variety of materials added to facilitate effective wound interfacings such as hydrogels, nanofibers and mats. MNPs can be incorporated or coated into common would dressing materials such as polyvinyl alcohol (PVA), chitosan, polycaprolactone (PCL) and cellulose [71,72]. Wound dressings incorporated with MNPs accelerate wound healing and possess significant antibacterial activity [71,73]. Applications of MNPs for wound healing have been widely explored, but they are still far from widespread commercialization and common clinical practice [7].

## 4. Metals and Metal Nanoparticles as Antimicrobials

While this review focuses on the wound healing aspects of metal nanoparticles, infection prevention is an important step in this process [74]. The use of metals as antimicrobials can be dated back to ancient civilizations including the Phoenicians, Greeks, Egyptians and Romans [75,76]. The past decade has seen an increase in the use of metals and metal nanoparticles for fighting infection [77,78,79,80]. As such, many metal nanoparticles are now commercially available and used in hospitals to coat wound dressings [78,81,82,83,84].

Metal ions have a broad range of antimicrobial activity with multiple cellular targets [77,85]. This is due to the metals unique chemistry which allows interactions with important functional groups in biological molecules [77]. The selectivity and strength of metal binding, which relates to its antimicrobial efficacy, follow trends seen in Hard-Soft-Acid-Base (HSAB) theory and ligand field theory [86,87]. Depending on the properties of the metal, different metals will target different functional groups on metabolites, proteins, nucleic acids, lipids and carbohydrates, any of which can cause deleterious cascading reactions in the cell [73]. Some of these effects include both direct and indirect membrane damage, altered membrane potential and solute transport, protein dysfunction and denaturation affecting metabolism, electron transport chain disruption, DNA damage and conformation changes, inhibition of DNA replication and repair, carbohydrate degradation and redox imbalance [88,89,90,91,92].

The production or propagation of reactive oxygen species (ROS) is another mechanism by which metals exert toxicity [77,93]. Certain metals are redox-active, with the ability to donate or accept electrons from another atom. Redox-active metals lead to reactive oxygen species by catalyzing Fenton reactions [93]. Other metals, not necessarily redox-active, may also cause ROS production through indirect means. Metals, such as silver and copper may interact with Fe-S clusters releasing free iron, a redox-active metal [94]. Inhibition of proteins or compounds involved in redox maintenance or of the electron transport chain may also indirectly lead to an increase in ROS levels [77]. Propagation of ROS in the cell can lead to protein, nucleic acid and membrane damage [95].

The antimicrobial activity of nanoparticles can be attributed to two aspects, the release of metal ions from the nanoparticle and the properties of the nanoparticle itself. Both the type of metal as well as the characteristics of the nanoparticle contribute to the observed antimicrobial activity. For example, smaller nanoparticles with a higher surface area to volume ratio are generally observed to be more toxic to bacteria [96,97,98] perhaps due to their ability to move through the cell membrane. The rate of metal dissolution also plays a large role in toxicity as nanoparticles with a faster rate of dissolution are often correlated with increased toxicity [99,100]. Overall, metal ions appear to be the main source of toxicity to the microbe with MNPs acting as efficient vehicles that concentrate metal ion release at the cell surface [80,99,101]. However, there are some exceptions such as TiO_2_ nanoparticle which acts through photocatalysis [102].

The first point of contact between the nanoparticle and the bacterial cell is either lipopolysaccharides for Gram-negative organisms [103] or teichoic and lipoteichoic acids and peptidoglycan for Gram-positives [104]. These structures typically carry negative charges allowing electrostatic interactions with positively charged MNPs and metal cations. These interactions can increase localized MNP and metal load locally, thus leading to a higher local dose to the bacterial cell. This localized load can lead to ROS production for secondary response and toxicity [90,105,106].

**Figure 1:**
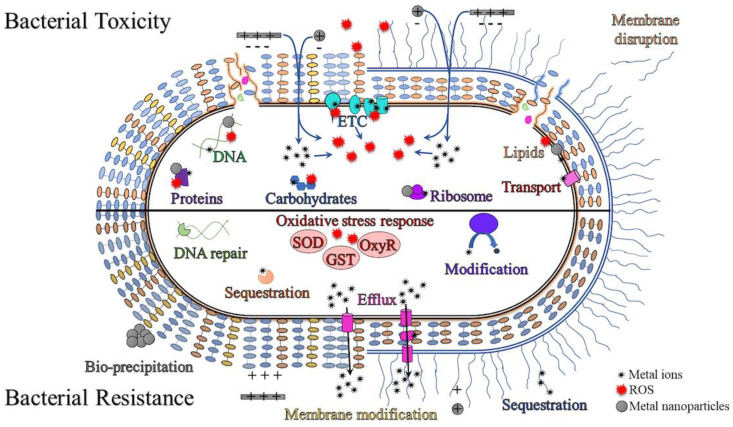
Summary of cellular targets (**top**) for metal and metal oxide nanoparticles and corresponding bacterial resistance mechanisms (**bottom**) in both Gram-positive (**left**) and Gram-negative (**right**) species. Before entry metal nanoparticles may produce reactive oxygen species (ROS), induce membrane fluidity and release metal ions [90]. Once inside the cell metal ions, nanoparticles or ROS may target the electron transport chain, ribosomes, DNA, proteins, lipids, carbohydrates and transport systems [90]. Bacteria respond by activating oxidative stress response proteins, DNA repair systems and metal efflux pumps [18,110,112]. Membrane modifications including an increase in flagellin, exopolysaccharides and changes to lipid composition can prevent metal entry by reducing electrostatic interactions and sequestering metal ions [18]. More resistant bacteria have also developed ways to modify metals into less toxic forms through reduction and/or bio-precipitation and nanoparticle aggregation [18,112,113].

The mechanisms of bacterial cell killing may or may not be related to the free metal. If the metal is released from dissociation of the NP, then the toxicity would occur similarly to the mechanisms described above for metal ions. However, some metal nanoparticles present killing properties very different from the source metal. For example, a non-redox active metal may become redox-active as a NP and thus, able to catalyze ROS production. For example, CuO or ZnO can produce ROS outside of the cell causing lipid peroxidation and cell damage [88,107]. If the metal nanoparticle can gain entry to the cell, further production of ROS may occur in addition, to interactions with other cell components including proteins, DNA/RNA, carbohydrates, lipids, ATP synthesis enzymes and the ribosome [90,108,109,110,111]. Though MNPs can target multiple cell processes, bacteria have developed ways to tolerate the metal ions released to mediate toxicity. Figure 1 provides an overview of the targets of metal nanoparticles within the bacterial cell as well as the resistance mechanisms employed by bacteria to deal with them.

### 4.1. Silver Nanoparticles

Silver NPs (AgNPs) or often referred to as colloidal silver, is the most well-known member of the MNP family which have been well explored for use in both wound healing and treating infection [31,114]. When silver is formulated as a NP it is often more effective for wound healing than silver ions alone [31]. Traditionally, silver was used to accelerate wound healing and other medical approaches, but its use was stunted with the discovery of antibiotics [4,115]. Although silver regained attention, its increased use has led to widespread wound bacteria resistance to silver nitrate and AgNPs [116]. Nevertheless, silver is being used more frequently due to the AMR era, especially in burn and wound treatment. For example, silver nitrate is still one of the best options for the treatment of chronic wounds [4]. It is well documented that AgNP based dressings accelerate wound healing by down-regulating metalloproteinases (a member of the collagenase enzyme group) [117]. Studies have shown that matrix metalloproteinases are essential for wound healing, but excess amounts of metalloproteinases will degrade peptide fibronectin and growth factors [7,117]. AgNPs, by enhancing apoptosis in cells and altering local matrix metalloproteinase secretion, play a key role in controlling inflammation in the injury area and accelerate the early phase of the healing process [117]. In addition, AgNP based dressings have anti-inflammatory potency by controlling the TNF- α expression level that leads to the prevention of necrosis in the wound [7,117].

### 4.2. Zinc Oxide Nanoparticles

Zinc (Zn) is a cofactor of several enzymes such as zinc-dependent matrix metalloproteinases that augment keratinocyte migration and auto debridement during wound repair [118]. Zn accelerates the wound healing process by various cellular and enzymatic activities. Studies demonstrated that dietary or hereditary Zn deficiency leads to delayed wound healing [118,119,120]. Both oral and local therapeutic usage of Zn are beneficial in wound healing [120]. However, topical administration of Zn salts seems to be preferable to oral prescription for wound treatment [118]. Zn reduces necrotic material and superinfections by collagenolytic activity, enhancing local immune response and stimulating epithelialization of wounds [118]. Zn accelerates the wound healing process by remaining at the injury site for an extended period of time [4,113]. Zinc oxide NPs (ZnO-NPs) in Unna boot (paste bandages) reduces inflammation and ulcer in the injured skin [118]. Hence, ZnO-NPs are frequently used in the cosmetic (as preservative) and pharmaceutical industries because of its excellent drying, anti-inflammatory and antimicrobial properties [4,121]. Furthermore, ZnO-NPs accelerated wound healing by releasing Zn ions and enhancing the keratinocyte migration in the injury site [4,121].

### 4.3. Gold Nanoparticles

Gold salt (tetrachloroaurate (III) trihydrate) has a very effective antimicrobial activity to a variety of pathogens growing in simulated wound fluid [122]. Gold NPs (AuNP) have been explored with considerable hope in wound healing as they have limited cytotoxicity, although their expense would limit their widespread use [123]. There are a few good examples of their use in wound healing. AuNPs along with a hydrogel of epigallocatechin gallate and α-lipoic acid are proven to be highly anti-inflammatory and potent antioxidants in wound healing [123,124,125]. AuNPs in combination with collagen display skin wound healing in a dose-dependent manner [126,127]. A study by Kim et al. demonstrated that hydrocolloid membrane coated with gold-nanoparticles significantly accelerated wound healing [128]. Another study by Volkova et al. showed the anti-oxidative and anti-microbial feature of AuNPs proving very effective in the regeneration of damaged collagen tissues and accelerating wound healing [129]. AuNPs accelerate the wound healing process through anti-inflammatory and anti-angiogenic activity via enhancing the secretion of vascular endothelial growth factor (VEGF) IL-12, IL-8 and TNF-α [124,125].

### 4.4. Titanium Dioxide Nanoparticles

Titanium dioxide (TiO_2_) is widely used in cosmetics and skin creams as a disinfectant. Early work showed that TiO_2_ nanowires had excellent efficacy compared to the NP form for skin infection control of *Staphylococcus aureus* [130]. TiO_2_ can be effective in wound healing because it has antibacterial effects against both Gram-negative and Gram-positive bacteria and cell growth properties [4]. The addition of TiO_2_ NPs decreased the dressing scaffold pore size, increased thermal stability and reduced swelling [131]. In addition, cytotoxicity tests on different cell lines such as osteoblast-like cells (MG-63), fibroblast human mesenchymal stem cells (hMSCs) and fibroblast cells (L929) showed that TiO_2_ NPs do not have cytotoxicity toward these cell lines [131]. Some studies used a combination of TiO_2_ with chitosan increasing the wound healing effect of chitosan [131,132]. TiO_2_ controls the haemorrhage by accelerating and enhanced clotting, at the same time, TiO_2_ supports platforms for cell adhesion, stem cells and growth of bone [131,132,133]. However, prolonged application of TiO_2_-NPs can lead to dermal cell toxicity and skin ageing caused by free radical generation, oxidative stress and collagen depletion [134,135,136]. Moreover, TiO_2_-NPs can penetrate the tissue through the skin and may be a human health risk [134]. Therefore, the use of TiO_2_-NPs should be avoided after UV irradiation, due to its photo-energy catalysis of free radicals, including hydroxyl radicals, inducing cell apoptosis and cytoskeletal dysfunction via DNA damage [134,136,137].

### 4.5. Metal Nanoparticle Potential Cytotoxicity

Though there are benefits of delivering metals in the form of NPs for wound healing applications, their organ and cytotoxicity should also be considered. Both metals and metal nanoparticles can cause adverse effects to the environment and human health, also affecting fish, algae and other mammals [138,139]. The cytotoxicity of the nanoparticle, like its antimicrobial activity, is related to its shape, size and formulation [140,141,142]. These characteristics can influence the cell uptake pathway, which in turn can lead to an increase or decrease in toxicity [143,144]. Changes to nanoparticle formulations as well as environmental factors will also affect the level of toxicity. For example, nanoparticles in the presence of surfactants or acidic environments can display greater toxicity [144,145]. Another study reported reduced toxicity for silver nanoparticles in the presence of dissolved organic matter or sulfides [146]. Recognizing the potential for the harmful side effects of engineered nanomaterials and instigating risk management is part of “safe by design” concepts and principles [147], that are now being implemented more frequently [148,149,150,151,152,153]. More research is also being conducted into the use of biogenic processes for nanoparticle production combined with new safety assessment procedures [148,149,150,151,152,153].

**Figure 2:**
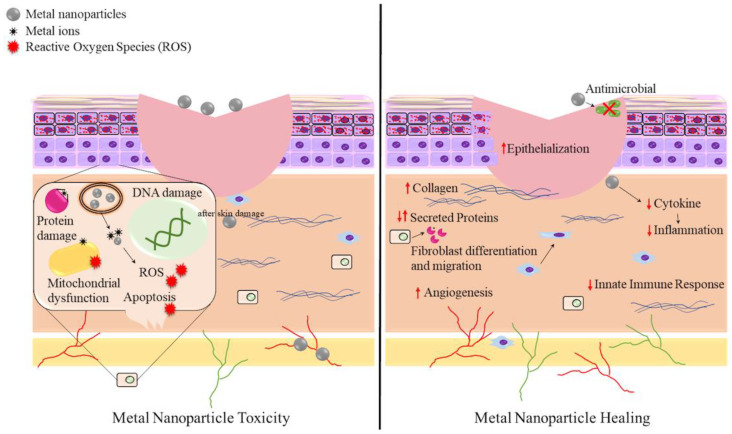
Comparison of nanoparticle toxicity (**left**) and healing potential (**right**) at the wound interface. Nanoparticles have been shown to damage skin cells such as keratinocytes and fibroblasts by inducing ROS after entry through endocytosis causing mitochondrial damage, genotoxicity and lipid peroxidation [158,159]. Similar damage can occur to other cells in addition to protein damage, altered signaling and metabolism, reduced adhesion and migration and induced cell death [144,160,161]. Wound healing properties of metal nanoparticles are displayed on the right. For example, AgNPs have been associated with cytokine modulation leading to reduced inflammation, innate immune response and scarring, while also inducing keratinocyte differentiation and migration [162]. AuNPs have also displayed anti-inflammatory properties in addition to increasing epithelization and collagen deposition [124,163]. They can also modulate proteins involved in the healing process such as superoxide dismutase, interleukin-8, interleukin-12, tumor necrosis factor-alpha, vascular endothelial growth factor and angiopoietin [164]. Other nanoparticles including copper, zinc oxide and titanium dioxide have shown similar effects while also having antimicrobial properties and increasing angiogenesis [80,121].

When examining the use of metal nanoparticles for wound healing a careful balance needs to be maintained between cytotoxicity and healing potential. As the main barrier of the human body is the skin, wounds which break this barrier allow more susceptibility to harmful substances. The close contact between the nanoparticle and the wound site allows both healing and potential for harm. Figure 2 summarizes both the healing properties and cytotoxicity of metal nanoparticles at the wound interface. In addition to specific effects at this interface, the ability of the nanoparticle to enter the bloodstream or lymphatic system must also be considered [154]. If allowed to traverse the body, antimicrobial nanoparticles may be toxic to various organs. Various metal nanoparticles have been shown to cause liver damage, kidney failure and neurological disorders, as well as affect immune function and reproduction in animal models [155,156]. Cell toxicity is primarily mediated by the production of ROS and release of metal ions after cell entry through endocytosis [141,144]. Additionally, there are dangers if the nanoparticles are able to cross the blood-brain barrier [157]. Therefore, nanoparticles with low cytotoxicity that are less able to cross animal cell membranes would be more useful for wound healing. This highlights the challenges in drug development to clinical use.

### 4.6. MNPs with Biomaterial in Wound Healing

Biomaterials such as chitosan, alginate, collagen and fibrin integrated into the forms of films, foams, wafers, hydrogels, sealants and composite dressings play a key role in hemostasis and wound healing in wound dressing materials [165]. They accelerate wound healing and skin development by fibrogenesis [4]. MNPs can be incorporated into the polymer network of the biomaterials to be used as a potential wound dressing material [4,166]. Several studies have shown that biomaterials incorporating different MNPs often enhance their wound healing potential while reducing harmful side effects [114,126,167,168]. Akturk et al. demonstrated that nanocomposite collagen scaffolds embedded with gold-NPs had excellent in vitro efficacy and biocompatibility to enhance wound healing [126]. Another study by Ahamed et al. showed faster wound healing with silver-NPs incorporated in cellulose and chitosan composites compared to the untreated control [167].

## 5. Concluding Comments and Perspective

Nanomaterials likely have limited medical applications for systemic bacterial control due to potential side effects. However, most antibiotics delivered systemically tend to have poor outcomes in wound care. Yet, topical antimicrobial formulations are overall more beneficial in wound care as this form of delivery provides a high dose locally. Regardless, antiseptics and disinfectants in topical wound applications can still have a variety of adverse effects. Although the bacteria causing the infection may be effectively targeted, there can still be delay in wound healing and other complications including damage to local tissue: cytotoxicity, contact dermatitis, anaphylaxis, rash, skin staining, additional pain/burning/itching; or organs: methemoglobinemia, liver toxicity, hyper/hypothyroidism, hemolysis, acidosis, pulmonary issues and renal damage [168]. For this reason, exploration of novel antimicrobials for wound treatments continues. Here, we see that NMs have potential, combining both wound healing and antimicrobial properties, with less potential side effects. Thus, we see a rapidly expanding field with many showing promising outcomes.

Combining different types of nanomaterials together is likely the future of this field. The ability of metal nanoparticles to target multiple bacterial biochemical processes makes them an appealing solution to avoid AMR development in a wound. Thus, combining metals into other NMs and types of polymers for effective fabrication methods for wound dressings. Bionanocomposites are still in their developmental infancy but development in this direction should lead to new products over the next decade. What is unfortunately inhibiting the field is the difficulty in comparing studies. Overall, systematic experiments are required that use defined pathogen indicator strains, mixed pathogen communities, clinical isolates, appropriate bacterial growth conditions (e.g., simulated wound fluid media) and follow up with appropriate animal wound models is critical to establish robust evidence of efficacy.

## Data Availability

No new data were created or analyzed in this study.

## References

[1] Kuehn B.M. (2007). Chronic Wound Care Guidelines IssuedChronic Wound Care Guidelines Issued. JAMA.

[2] Wang W., Lu K.-J., Yu C.-H., Huang Q.-L., Du Y.-Z. (2019). Nano-drug delivery systems in wound treatment and skin regeneration. J. Nanobiotechnol..

[3] Jahromi M.A.M., Zangabad P.S., Basri S.M.M., Zangabad K.S., Ghamarypour A., Aref A.R., Karimi M., Hamblin M.R. (2018). Nanomedicine and advanced technologies for burns: Preventing infection and facilitating wound healing. Adv. Drug Deliv. Rev..

[4] Deepachitra R., Lakshmi R.P., Sivaranjani K., Chandra J.H., Sastry T.P. (2015). Nanoparticles embedded biomaterials in wound treatment: A review. J. Chem. Pharm. Sci..

[5] Akers K.S., Wenke J.C., Murray C.K., Williams D. (2019). Biofilms and Wound Infection Research in the US Military. Targeting Biofilms in Translational Research, Device Development, and Industrial Sectors.

[6] O’Neill J. (2014). Antimicrobial resistance: Tackling a Crisis for the Health and Wealth of Nations. Wellcome Collection.

[7] Kalantari K., Mostafavi E., Afifi A.M., Izadiyan Z., Jahangirian H., Rafiee-Moghaddam R., Webster T.J. (2020). Wound dressings functionalized with silver nanoparticles: Promises and pitfalls. Nanoscale.

[8] Wang E.T., Sandberg R., Luo S., Khrebtukova I., Zhang L., Mayr C., Kingsmore S.F., Schroth G.P., Burge C.B. (2008). Alternative isoform regulation in human tissue transcriptomes. Nature.

[9] Shanmugasundaram T., Radhakrishnan M., Gopikrishnan V., Kadirvelu K., Balagurunathan R. (2017). In vitro antimicrobial and in vivo wound healing effect of actinobacterially synthesised nanoparticles of silver, gold and their alloy. RSC Adv..

[10] Stoica A.E., Chircov C., Grumezescu A.M. (2020). Nanomaterials for Wound Dressings: An Up-to-Date Overview. Molecules.

[11] Allen T.M., Cullis P.R. (2013). Liposomal drug delivery systems: From concept to clinical applications. Adv. Drug Deliv. Rev..

[12] Bespalova Y., Kwon D., Vasanthan N. (2017). Surface modification and antimicrobial properties of cellulose nanocrystals. J. Appl. Polym. Sci..

[13] Gehring J., Schleheck D., Trepka B., Polarz S. (2014). Mesoporous Organosilica Nanoparticles Containing Superacid and Click Functionalities Leading to Cooperativity in Biocidal Coatings. ACS Appl. Mater. Interfaces.

[14] Seisenbaeva G.A., Fromell K., Vinogradov V.V., Terekhov A.N., Pakhomov A.V., Nilsson E.K., Ekdahl K.N., Vinogradov V.V., Kessler V.G. (2017). Dispersion of TiO_2_ nanoparticles improves burn wound healing and tissue regeneration through specific interaction with blood serum proteins. Sci. Rep..

[15] Nam G., Rangasamy S., Purushothaman B., Song J.M. (2015). The Application of Bactericidal Silver Nanoparticles in Wound Treatment. Nanomater. Nanotechnol..

[16] Lee N.-Y., Ko W.-C., Hsueh P.-R. (2019). Nanoparticles in the Treatment of Infections Caused by Multidrug-Resistant Organisms. Front. Pharmacol..

[17] Huh A.J., Kwon Y.J. (2011). Nanoantibiotics: A new paradigm for treating infectious diseases using nanomaterials in the antibiotics resistant era. J. Control. Release.

[18] Niño-Martínez N., Orozco M.F.S., Martínez-Castañón G.-A., Méndez F.T., Ruiz F. (2019). Molecular Mechanisms of Bacterial Resistance to Metal and Metal Oxide Nanoparticles. Int. J. Mol. Sci..

[19] Sánchez-López E., Gomes D., Esteruelas G., Bonilla L., Lopez-Machado A.L., Galindo R., Cano A., Espina M., Ettcheto M., Camins A. (2020). Metal-Based Nanoparticles as Antimicrobial Agents: An Overview. Nanomaterials.

[20] Arora N., Thangavelu K., Karanikolos G.N. (2020). Bimetallic Nanoparticles for Antimicrobial Applications. Front. Chem..

[21] Shkodenko L., Kassirov I., Koshel E. (2020). Metal Oxide Nanoparticles Against Bacterial Biofilms: Perspectives and Limitations. Microorganisms.

[22] McDonnell G., Russell A.D. (1999). Antiseptics and Disinfectants: Activity, Action, and Resistance. Clin. Microbiol. Rev..

[23] Kujath P., Michelsen A. (2008). Wounds–From physiology to wound dressing. Dtsch. Ärzteblatt Int..

[24] Wilkins R.G., Unverdorben M. (2013). Wound cleaning and wound healing: A concise review. Adv. Skin Wound Care.

[25] Levy S.M., Holzmann-Pazgal G., Lally K.P., Davis K., Kao L.S., Tsao K. (2013). Quality Check of a Quality Measure: Surgical Wound Classification Discrepancies Impact Risk-Stratified Surgical Site Infection Rates in Pediatric Appendicitis. J. Am. Coll. Surg..

[26] Onyekwelu I., Yakkanti R., Protzer L., Pinkston C.M., Tucker C., Seligson D. (2017). Surgical wound classification and surgical site infections in the orthopaedic patient. J. Am. Acad. Orthop. Surg. Glob. Res. Rev..

[27] Upton D., Solowiej K., Hender C., Woodyatt K.Y. (2012). Stress and pain associated with dressing change in patients with chronic wounds. J. Wound Care.

[28] Gurtner G.C., Werner S., Barrandon Y., Longaker M.T. (2008). Wound repair and regeneration. Nat. Cell Biol..

[29] Pastar I., Stojadinovic O., Yin N.C., Ramirez H., Nusbaum A.G., Sawaya A., Patel S.B., Khalid L., Isseroff R.R., Tomic-Canic M. (2014). Epithelialization in Wound Healing: A Comprehensive Review. Adv. Wound Care.

[30] Debone H.S., Lopes P.S., Severino P., Yoshida C.M.P., Souto E.B., da Silva C.F. (2019). Chitosan/Copaiba oleoresin films for would dressing application. Int. J. Pharm..

[31] Souto E.B., Ribeiro A.F., Ferreira M.I., Teixeira M.C., Shimojo A.A.M., Soriano J.L., Naveros B.C., Durazzo A., Lucarini M., Souto S.B. (2020). New Nanotechnologies for the Treatment and Repair of Skin Burns Infections. Int. J. Mol. Sci..

[32] Gao W., Chen Y., Zhang Y., Zhang Q., Zhang L. (2018). Nanoparticle-based local antimicrobial drug delivery. Adv. Drug Deliv. Rev..

[33] Lipsky B.A., Hoey C. (2009). Topical Antimicrobial Therapy for Treating Chronic Wounds. Clin. Infect. Dis..

[34] Malekzad H., Mirshekari H., Zangabad P.S., Basri S.M.M., Baniasadi F., Aghdam M.S., Karimi M., Hamblin M.R. (2018). Plant protein-based hydrophobic fine and ultrafine carrier particles in drug delivery systems. Crit. Rev. Biotechnol..

[35] Piacenza E., Presentato A., Turner R.J. (2018). Stability of biogenic metal(loid) nanomaterials related to the colloidal stabilization theory of chemical nanostructures. Crit. Rev. Biotechnol..

[36] Rakhmetova A.A., Alekseeva T.P., Bogoslovskaya O.A., Leipunskii I.O., Ol’Khovskaya I.P., Zhigach A.N., Glushchenko N.N. (2010). Wound-healing properties of copper nanoparticles as a function of physicochemical parameters. Nanotechnol. Russ..

[37] Yu X., Trase I., Ren M., Duval K., Guo X., Chen Z. (2016). Design of Nanoparticle-Based Carriers for Targeted Drug Delivery. J. Nanomater..

[38] Zhao M.-X., Zeng E.-Z., Zhu B.-J. (2015). The Biological Applications of Inorganic Nanoparticle Drug Carriers. ChemNanoMat.

[39] Angelova A., Garamus V.M., Angelov B., Tian Z., Li Y., Zou A. (2017). Advances in structural design of lipid-based nanoparticle carriers for delivery of macromolecular drugs, phytochemicals and anti-tumor agents. Adv. Colloid Interface Sci..

[40] Selvarajan V., Obuobi S., Ee P.L.R. (2020). Silica Nanoparticles—A Versatile Tool for the Treatment of Bacterial Infections. Front. Chem..

[41] Kumar R., Mondal K., Panda P.K., Kaushik A., Abolhassani R., Ahuja R., Rubahn H.-G., Mishra Y.K. (2020). Core–shell nanostructures: Perspectives towards drug delivery applications. J. Mater. Chem. B.

[42] Balaure P.C., Grumezescu A.M. (2020). Recent Advances in Surface Nanoengineering for Biofilm Prevention and Control. Part I: Molecular Basis of Biofilm Recalcitrance. Passive Anti-Biofouling Nanocoatings. Nanomaterials.

[43] Balaure P.C., Grumezescu A.M. (2020). Recent Advances in Surface Nanoengineering for Biofilm Prevention and Control. Part II: Active, Combined Active and Passive, and Smart Bacteria-Responsive Antibiofilm Nanocoatings. Nanomaterials.

[44] Kumar R., Kumar V.B., Gedanken A. (2020). Sonochemical synthesis of carbon dots, mechanism, effect of parameters, and catalytic, energy, biomedical and tissue engineering applications. Ultrason Sonochem..

[45] Li P., Liu S., Yang X., Du S., Tang W., Cao W., Zhou J., Gong X., Xing X. (2021). Low-drug resistance carbon quantum dots decorated injectable self-healing hudrogel with potent antibiofilm property and cutaneous wound healing. Chem. Eng. J..

[46] Kale S.N., Kitture R., Ghosh S., Chopade B.A., Yakhmi J.V. (2017). Nanomaterials as Enhanced Antimicrobial Agent/Activity-Enhancer for Transdermal Applications: A Review. Antimicrobial Nanoarchitectonics.

[47] Kitture R., Ghosh S. (2019). Hybrid Nanostructures for In Vivo Imaging. Hybrid Nanostructures for Cancer Theranostics.

[48] Chen G., Wu Y., Yu D., Li R., Luo W., Ma G., Zhang C. (2018). Isoniazid-loaded chitosan/carbon nanotubes microspheres promote secondary wound healing of bone tuberculosis. J. Biomater. Appl..

[49] Kittana N., Assali M., Abu-Rass H., Lutz S., Hindawi R., Ghannam L., Zakarneh M., Mosa A. (2018). Enhancement of wound healing by single-wall/multi-wall carbon nanotubes complexed with chitosan. Int. J. Nanomed..

[50] Santos J.C.C., Mansur A.A.P., Ciminelli V.S.T., Mansur H.S. (2014). Nanocomposites of Poly(Vinyl Alcohol)/Functionalized-Multiwall Carbon Nanotubes Conjugated With Glucose Oxidase for Potential Application as Scaffolds in Skin Wound Healing. Int. J. Polym. Mater..

[51] Slaughter B.V., Khurshid S.S., Fisher O.Z., Khademhosseini A., Peppas N.A. (2009). Hydrogels in Regenerative Medicine. Adv. Mater..

[52] Ravanbakhsh H., Bao G., Mongeau L. (2020). Carbon nanotubes promote cell migration in hydrogels. Sci. Rep..

[53] Ghuge A.D., Shirode A.R., Kadam V.J. (2017). Graphene: A Comprehensive Review. Curr. Drug Targets.

[54] Ghosh S., Sanghavi S., Sancheti P., Balakrishnan P., Sreekala M.S., Thomas S. (2018). Metallic Biomaterial for Bone Support and Replacement. Fundamental Biomaterials: Metals.

[55] Fan Z., Liu B., Wang J., Zhang S., Lin Q., Gong P., Ma L., Yang S. (2014). A Novel Wound Dressing Based on Ag/Graphene Polymer Hydrogel: Effectively Kill Bacteria and Accelerate Wound Healing. Adv. Funct. Mater..

[56] Fu J., Zhang Y., Chu J., Wang X., Yan W., Zhang Q., Liu H. (2019). Reduced Graphene Oxide Incorporated Acellular Dermal Composite Scaffold Enables Efficient Local Delivery of Mesenchymal Stem Cells for Accelerating Diabetic Wound Healing. ACS Biomater. Sci. Eng..

[57] Rehman S.R.U., Augustine R., Zahid A.A., Ahmed R., Tariq M., Hasan A. (2019). Reduced Graphene Oxide Incorporated GelMA Hydrogel Promotes Angiogenesis For Wound Healing Applications. Int. J. Nanomed..

[58] Tang P., Lu H., Pengfei L., Zhanrong J., Kefeng W., Hongping Z., Hui T., Tailin G., Xiong L. (2019). Mussel-Inspired Electroactive and Antioxidative Scaffolds with Incorporation of Polydopamine-Reduced Graphene Oxide for Enhancing Skin Wound Healing. ACS Appl. Mater. Interfaces.

[59] Lipinski C.A. (2004). Lead- and drug-like compounds: The rule-of-five revolution. Drug Discov. Today Technol..

[60] Mengoni T., Adrian M., Pereira S., Santos-Carballal B., Kaiser M., Goycoolea F.M. (2017). A Chitosan—Based Liposome Formulation Enhances the In Vitro Wound Healing Efficacy of Substance P Neuropeptide. Pharmaceutics.

[61] Ternullo S., Werning L.V.S., Holsæter A.M., Škalko-Basnet N. (2019). Curcumin-In-Deformable Liposomes-In-Chitosan-Hydrogel as a Novel Wound Dressing. Pharmaceutics.

[62] Cui M.-D., Pan Z.-H., Pan L.-Q. (2017). Danggui Buxue Extract-Loaded Liposomes in Thermosensitive Gel Enhance In Vivo Dermal Wound Healing via Activation of the VEGF/PI3K/Akt and TGF-β/Smads Signaling Pathway. Evid. Based Complement. Altern. Med..

[63] Harrison J.J., Ceri H., Turner R.J. (2007). Multimetal resistance and tolerance in microbial biofilms. Nat. Rev. Genet..

[64] Quester K., Avalos-Borja M., Castro-Longoria E. (2013). Biosynthesis and microscopic study of metallic nanoparticles. Micron.

[65] Schröfel A., Kratošová G., Šafařík I., Šafaříková M., Raška I., Shor L.M. (2014). Applications of biosynthesized metallic nanoparticles–A review. Acta Biomater..

[66] Zhao L., Ashraf M.A. (2015). Influence of silver-hydroxyapatite nanocomposite coating on biofilm formation of joint prosthesis and its mechanism. West Indian Med. J..

[67] Zonaro E., Lampis S., Turner R.J., Qazi S.J.S., Vallini G. (2015). Biogenic selenium and tellurium nanoparticles synthesized by environmental microbial isolates efficaciously inhibit bacterial planktonic cultures and biofilms. Front. Microbiol..

[68] Chatzimitakos T., Stalikas C.D. (2016). Qualitative Alterations of Bacterial Metabolome after Exposure to Metal Nanoparticles with Bactericidal Properties: A Comprehensive Workflow Based on 1H NMR, UHPLC-HRMS, and Metabolic Databases. J. Proteome Res..

[69] Wang L., Hu C., Shao L. (2017). The antimicrobial activity of nanoparticles: Present situation and prospects for the future. Int. J. Nanomed..

[70] Piacenza E., Presentato A., Zonaro E., Lemire J.A., Demeter M., Vallini G., Turner R.J., Lampis S. (2017). Antimicrobial activity of biogenically produced spherical Se-nanomaterials embedded in organic material against Pseudomonas aeruginosa and Staphylococcus aureus strains on hydroxyapatite-coated surfaces. Microb. Biotechnol..

[71] Khorasani M.T., Joorabloo A., Moghaddam A., Shamsi H., MansooriMoghadam Z. (2018). Incorporation of ZnO nanoparticles into heparinised polyvinyl alcohol/chitosan hydrogels for wound dressing application. Int. J. Biol. Macromol..

[72] Tao J., Xu X., Liu H., Jiang X., Mao J., Gou M. (2020). A nanoparticle-functionalized wound dressing device for toxin neutralization. Mater. Des..

[73] Li Q., Lu F., Zhou G., Yu K., Lu B., Xiao Y., Dai F., Wu D., Lan G. (2017). Silver Inlaid with Gold Nanoparticle/Chitosan Wound Dressing Enhances Antibacterial Activity and Porosity, and Promotes Wound Healing. Biomacromolecules.

[74] Li Z., Knetsch M. (2018). Antibacterial strategies for wound dressing: Preventing infection and stimulating healing. Curr. Pharm. Des..

[75] Dollwet H., Sorenson J. (1985). Historic uses of copper compounds in medicine. Trace Elem. Med..

[76] Barillo D.J., Marx D.E. (2014). Silver in medicine: A brief history BC 335 to present. Burns.

[77] Lemire J.A., Harrison J.J., Turner R.J. (2013). Antimicrobial activity of metals: Mechanisms, molecular targets and applications. Nat. Rev. Genet..

[78] Ficai D., Oprea O., Ficai A., Holban A. (2014). Metal Oxide Nanoparticles: Potential Uses in Biomedical Applications. Curr. Proteom..

[79] Turner R.J. (2017). Metal-based antimicrobial strategies. Microb. Biotechnol..

[80] Khezerlou A., Alizadeh-Sani M., Azizi-Lalabadi M., Ehsani A. (2018). Nanoparticles and their antimicrobial properties against pathogens including bacteria, fungi, parasites and viruses. Microb. Pathog..

[81] Khan S.T., Musarrat J., Al-Khedhairy A.A. (2016). Countering drug resistance, infectious diseases, and sepsis using metal and metal oxides nanoparticles: Current status. Colloids Surf. B Biointerfaces.

[82] Babushkina I.V., Gladkova E.V., Belova S.V., Norkin I.A. (2017). Application of Preparations Containing Copper Nanoparticles for the Treatment of Experimental Septic Wounds. Bull. Exp. Biol. Med..

[83] Kwiatkowska A., Granicka L.H., Grzeczkowicz A., Stachowiak R., Bącal P., Sobczak K., Darowski M., Kozarski M., Bielecki J. (2018). Gold Nanoparticle-Modified Poly(vinyl chloride) Surface with Improved Antimicrobial Properties for Medical Devices. J. Biomed. Nanotechnol..

[84] Vijayakumar V., Samal S.K., Mohanty S., Nayak S.K. (2019). Recent advancements in biopolymer and metal nanoparticle-based materials in diabetic wound healing management. Int. J. Biol. Macromol..

[85] Lemire J.A., Turner R.J. (2016). Mechanisms Underlying the Antimicrobial Capacity of Metals. Stress and Environmental Regulation of Gene Expression and Adaptation in Bacteria.

[86] Griffith J.S., Orgel L.E. (1957). Ligand-field theory. Q. Rev. Chem. Soc..

[87] Jones M.M., Vaughn W.K. (1978). HSAB theory and acute metal ion toxicity and detoxification processes. J. Inorg. Nucl. Chem..

[88] Meghana S., Kabra P., Chakraborty S., Padmavathy N. (2015). Understanding the pathway of antibacterial activity of copper oxide nanoparticles. RSC Adv..

[89] Li H., Gao Y., Li C., Ma G., Shang Y., Sun Y. (2016). A comparative study of the antibacterial mechanisms of silver ion and silver nanoparticles by Fourier transform infrared spectroscopy. Vib. Spectrosc..

[90] Slavin Y.N., Asnis J., Häfeli U.O., Bach H. (2017). Metal nanoparticles: Understanding the mechanisms behind antibacterial activity. J. Nanobiotechnol..

[91] Kędziora A., Speruda M., Krzyżewska E., Rybka J., Łukowiak A., Bugla-Płoskońska G. (2018). Similarities and differences between silver ions and silver in nanoforms as antibacterial agents. Int. J. Mol. Sci..

[92] Kadiyala U., Turali-Emre E.S., Bahng J.H., Kotov N.A., Vanepps J.S. (2018). Unexpected insights into antibacterial activity of zinc oxide nanoparticles against methicillin resistant Staphylococcus aureus (MRSA). Nanoscale.

[93] Stohs S.J., Bagchi D. (1995). Oxidative mechanisms in the toxicity of metal ions. Free Radic. Biol. Med..

[94] Xu F.F., Imlay J.A. (2012). Silver(I), Mercury(II), Cadmium(II), and Zinc(II) Target Exposed Enzymic Iron-Sulfur Clusters when They Toxify Escherichia coli. Appl. Environ. Microbiol..

[95] Imlay J.A. (2003). Pathways of Oxidative Damage. Annu. Rev. Microbiol..

[96] Azam A., Ahmed A.S., Oves M., Khan M.S., Memic A. (2012). Size-dependent antimicrobial properties of CuO nanoparticles against Gram-positive and -negative bacterial strains. Int. J. Nanomed..

[97] Kumari M., Pandey S., Giri V.P., Bhattacharya A., Shukla R., Mishra A., Nautiyal C. (2017). Tailoring shape and size of biogenic silver nanoparticles to enhance antimicrobial efficacy against MDR bacteria. Microb. Pathog..

[98] Kaushik M., Niranjan R., Thangam R., Madhan B., Pandiyarasan V., Ramachandran C., Oh D.-H., Venkatasubbu G.D. (2019). Investigations on the antimicrobial activity and wound healing potential of ZnO nanoparticles. Appl. Surf. Sci..

[99] Sowa-Söhle E.N., Schwenke A., Wagener P., Weiss A., Wiegel H., Sajti C.L., Haverich A., Barcikowski S., Loos A. (2013). Antimicrobial efficacy, cytotoxicity, and ion release of mixed metal (Ag, Cu, Zn, Mg) nanoparticle polymer composite implant material. BioNanoMaterials.

[100] Kubo A.-L., Capjak I., Vrček I.V., Bondarenko O.M., Kurvet I., Vija H., Ivask A., Kasemets K., Kahru A. (2018). Antimicrobial potency of differently coated 10 and 50 nm silver nanoparticles against clinically relevant bacteria Escherichia coli and Staphylococcus aureus. Colloids Surf. B Biointerfaces.

[101] Dorobantu L.S., Fallone C., Noble A.J., Veinot J.G.C., Ma G., Goss G.G., Burrell R.E. (2015). Toxicity of silver nanoparticles against bacteria, yeast, and algae. J. Nanopart. Res..

[102] Foster H.A., Ditta I.B., Varghese S., Steele A. (2011). Photocatalytic disinfection using titanium dioxide: Spectrum and mechanism of antimicrobial activity. Appl. Microbiol. Biotechnol..

[103] Jacobson K.H., Gunsolus I.L., Kuech T.R., Troiano J.M., Melby E.S., Lohse S.E., Hu D., Chrisler W.B., Murphy C.J., Orr G. (2015). Lipopolysaccharide Density and Structure Govern the Extent and Distance of Nanoparticle Interaction with Actual and Model Bacterial Outer Membranes. Environ. Sci. Technol..

[104] Pajerski W., Ochonska D., Brzychczy-Wloch M., Indyka P., Jarosz M., Golda-Cepa M., Sojka Z., Kotarba A. (2019). Attachment efficiency of gold nanoparticles by Gram-positive and Gram-negative bacterial strains governed by surface charges. J. Nanopart. Res..

[105] Kora A.J., Arunachalam J. (2010). Assessment of antibacterial activity of silver nanoparticles on Pseudomonas aeruginosa and its mechanism of action. World J. Microbiol. Biotechnol..

[106] Ivask A., Elbadawy A., Kaweeteerawat C., Boren D., Fischer H., Ji Z., Chang C.H., Liu R., Tolaymat T., Telesca D. (2014). Toxicity Mechanisms in Escherichia coli Vary for Silver Nanoparticles and Differ from Ionic Silver. ACS Nano.

[107] Jiang Y., Zhang L., Wen D., Ding Y. (2016). Role of physical and chemical interactions in the antibacterial behavior of ZnO nanoparticles against E. coli. Mater. Sci. Eng. C.

[108] Cui Y., Zhao Y., Tian Y., Zhang W., Lü X., Jiang X. (2012). The molecular mechanism of action of bactericidal gold nanoparticles on Escherichia coli. Biomaterials.

[109] Raghunath A., Perumal E. (2017). Metal oxide nanoparticles as antimicrobial agents: A promise for the future. Int. J. Antimicrob. Agents.

[110] Tiwari V., Mishra N., Gadani K., Solanki P.S., Shah N.A., Tiwari M. (2018). Mechanism of Anti-bacterial Activity of Zinc Oxide Nanoparticle Against Carbapenem-Resistant Acinetobacter baumannii. Front. Microbiol..

[111] Ortiz-Benítez E.A., Velázquez-Guadarrama N., Figueroa N.V.D., Quezada H., Olivares-Trejo J.D.J. (2019). Antibacterial mechanism of gold nanoparticles on Streptococcus pneumoniae. Metallomics.

[112] Hobman J.L., Crossman L.C. (2015). Bacterial antimicrobial metal ion resistance. J. Med. Microbiol..

[113] Muller M., Merrett N.D. (2014). Pyocyanin Production by Pseudomonas aeruginosa Confers Resistance to Ionic Silver. Antimicrob. Agents Chemother..

[114] Kumar S.S.D., Rajendran N.K., Houreld N.N., Abrahamse H. (2018). Recent advances on silver nanoparticle and biopolymer-based biomaterials for wound healng applications. Int. J. Biol. Macromol..

[115] Wilkinson L., White R., Chipman J. (2011). Silver and nanoparticles of silver in wound dressings: A review of efficacy and safety. J. Wound Care.

[116] Panáček A., Kvítek L., Smékalová M., Večeřová R., Kolář M., Röderová M., Dyčka F., Šebela M., Prucek R., Tomanec O. (2018). Bacterial resistance to silver nanoparticles and how to overcome it. Nat. Nanotechnol..

[117] Wright J.B., Lam K., Buret A.G., Olson M.E., Burrell R.E. (2002). Early healing events in a porcine model of contaminated wounds: Effects of nanocrystalline silver on matrix metalloproteinases, cell apoptosis, and healing. Wound Repair Regen..

[118] Lansdown A.B.G., Mirastschijski U., Stubbs N., Scanlon E., Ågren M.S. (2007). Zinc in wound healing: Theoretical, experimental, and clinical aspects. Wound Repair Regen..

[119] Lim Y., Levy M., Bray T.M. (2004). Dietary Zinc Alters Early Inflammatory Responses during Cutaneous Wound Healing in Weanling CD-1 Mice. J. Nutr..

[120] Stechmiller J.K. (2010). Understanding the Role of Nutrition and Wound Healing. Nutr. Clin. Pr..

[121] Rajendran N.K., Kumar S.S.D., Houreld N.N., Abrahamse H. (2018). A review on nanoparticle based treatment for wound healing. J. Drug Deliv. Sci. Technol..

[122] Pormohammad A., Turner R.J. (2020). Silver Antibacterial Synergism Activities with Eight Other Metal(loid)-Based Antimicrobials against *Escherichia coli*, *Pseudomonas aeruginosa*, and *Staphylococcus aureus*. Antibiotics.

[123] Pivodová V., Franková J., Galandáková A., Ulrichová J. (2015). In Vitro AuNPs’ Cytotoxicity and Their Effect on Wound Healing. Nanobiomedicine.

[124] Leu J.-G., Chen S.-A., Chen H.-M., Wu W.-M., Hung C.-F., Yao Y.-D., Tu C.-S., Liang Y.-J. (2012). The effects of gold nanoparticles in wound healing with antioxidant epigallocatechin gallate and α-lipoic acid. Nanomed. Nanotechnol. Biol. Med..

[125] Ovais M., Ahmad I., Khalil A.T., Mukherjee S., Javed R., Ayaz M., Raza A., Shinwari Z.K. (2018). Wound healing applications of biogenic colloidal silver and gold nanoparticles: Recent trends and future prospects. Appl. Microbiol. Biotechnol..

[126] Akturk O., Kismet K., Yasti A.C., Kuru S., Duymus M.E., Kaya F., Caydere M., Hucumenoglu S., Keskin D. (2016). Collagen/gold nanoparticle nanocomposites: A potential skin wound healing biomaterial. J. Biomater. Appl..

[127] Akturk O., Kismet K., Yasti A.C., Kuru S., Duymus M.E., Kaya F., Caydere M., Hucumenoglu S., Keskin D. (2016). Wet electrospun silk fibroin/gold nanoparticle 3D matrices for wound healing applications. RSC Adv..

[128] Kim J.E., Lee J., Jang M., Kwak M.H., Go J., Kho E.K., Song S.H., Sung J.E., Lee J., Hwang D.Y. (2015). Accelerated healing of cutaneous wounds using phytochemically stabilized gold nanoparticle deposited hydrocolloid membranes. Biomater. Sci..

[129] Volkova N., Yukhta M., Pavlovich O., Goltsev A. (2016). Application of Cryopreserved Fibroblast Culture with Au Nanoparticles to Treat Burns. Nanoscale Res. Lett..

[130] Nataraj N., Anjusree G.S., Madhavan A.A., Priyanka P., Sankar D., Nisha N., Lakshmi S.V., Jayakumar R., Balakrishnan A., Biswas R. (2014). Synthesis and anti-staphylococcal activity of TiO_2_ nanoparticles and nanowires in ex vivo porcine skin model. J. Biomed. Nanotechnol..

[131] Bui V.K.H., Park D., Lee Y.-C. (2017). Chitosan Combined with ZnO, TiO_2_ and Ag Nanoparticles for Antimicrobial Wound Healing Applications: A Mini Review of the Research Trends. Polymers.

[132] Peng L., Eltgroth M.L., LaTempa T.J., Grimes C.A., Desai T.A. (2009). The effect of TiO_2_ nanotubes on endothelial function and smooth muscle proliferation. Biomaterials.

[133] Brammer K.S., Oh S., Gallagher J.O., Jin S. (2008). Enhanced Cellular Mobility Guided by TiO_2_ Nanotube Surfaces. Nano Lett..

[134] Wamer W.G., Yin J.-J., Wei R.R. (1997). Oxidative Damage to Nucleic Acids Photosensitized by Titanium Dioxide. Free Radic. Biol. Med..

[135] Wu J., Liu W., Xue C., Zhou S., Lan F., Bi L., Xu H., Yang X., Zeng F.-D. (2009). Toxicity and penetration of TiO_2_ nanoparticles in hairless mice and porcine skin after subchronic dermal exposure. Toxicol. Lett..

[136] Naves L.B., Almeida L. (2015). Wound Healing Dressing and Some Composites Such as Zeolite, TiO_2_, Chitosan and PLGA: A Review. Int. J. Miner. Metall. Mater..

[137] Herrling T., Jung K., Fuchs J. (2006). Measurements of UV-generated free radicals/reactive oxygen species (ROS) in skin. Spectrochim. Acta Part A Mol. Biomol. Spectrosc..

[138] Bondarenko O., Juganson K., Ivask A., Kasemets K., Mortimer M., Kahru A. (2013). Toxicity of Ag, CuO and ZnO nanoparticles to selected environmentally relevant test organisms and mammalian cells in vitro: A critical review. Arch. Toxicol..

[139] Yue Y., Li X., Sigg L., Marc J.F.S., Pillai S., Behra R., Schirmer K. (2017). Interaction of silver nanoparticles with algae and fish cells: A side by side comparison. J. Nanobiotechnol..

[140] Park M.V., Neigh A.M., Vermeulen J.P., De La Fonteyne L.J., Verharen H.W., Briedé J.J., Van Loveren H., De Jong W.H. (2011). The effect of particle size on the cytotoxicity, inflammation, developmental toxicity and genotoxicity of silver nanoparticles. Biomaterials.

[141] Seitz F., Rosenfeldt R.R., Storm K., Metreveli G., Schaumann G.E., Schulz R., Bundschuh M. (2015). Effects of silver nanoparticle properties, media pH and dissolved organic matter on toxicity to Daphnia magna. Ecotoxicol. Environ. Saf..

[142] Sultana S., Djaker N., Boca-Farcau S., Salerno M., Charnaux N., Astilean S., Hlawaty H., De La Chapelle M.L. (2015). Comparative toxicity evaluation of flower-shaped and spherical gold nanoparticles on human endothelial cells. Nanotechnology.

[143] Guarnieri D., Sabella S., Muscetti O., Belli V., Malvindi M.A., Fusco S., De Luca E., Pompa P.P., Netti P.A. (2014). Transport across the cell-membrane dictates nanoparticle fate and toxicity: A new paradigm in nanotoxicology. Nanoscale.

[144] Sabella S., Carney R.P., Brunetti V., Malvindi M.A., Al-Juffali N., Vecchio G., Janes S.M., Bakr O.M., Cingolani R., Stellacci F. (2014). A general mechanism for intracellular toxicity of metal-containing nanoparticles. Nanoscale.

[145] Ginzburg A.L., Truong L., Tanguay R.L., Hutchison J.E. (2018). Synergistic Toxicity Produced by Mixtures of Biocompatible Gold Nanoparticles and Widely Used Surfactants. ACS Nano.

[146] Lee S.-W., Park S.-Y., Kim Y., Im H., Choi J. (2016). Effect of sulfidation and dissolved organic matters on toxicity of silver nanoparticles in sediment dwelling organism, Chironomus riparius. Sci. Total Environ..

[147] Van De Poel I., Robaey Z. (2017). Safe-by-Design: From Safety to Responsibility. NanoEthics.

[148] Pokhrel L.R., Silva T., Dubey B., El Badawy A.M., Tolaymat T.M., Scheuerman P.R. (2012). Rapid screening of aquatic toxicity of several metal-based nanoparticles using the MetPLATE TM bioassay. Sci. Total Environ..

[149] Kar S., Gajewicz A., Roy K., Leszczynski J., Puzyn T. (2016). Extrapolating between toxicity endpoints of metal oxide nanoparticles: Predicting toxicity to Escherichia coli and human keratinocyte cell line (HaCaT) with Nano-QTTR. Ecotoxicol. Environ. Saf..

[150] Van Pomeren M., Peijnenburg W.J.G.M., Brun N.R., Vijver M.G. (2017). A Novel Experimental and Modelling Strategy for Nanoparticle Toxicity Testing Enabling the Use of Small Quantities. Int. J. Environ. Res. Public Heal..

[151] Bastús N.G., Puntes V. (2018). Nanosafety: Towards Safer Nanoparticles by Design. Curr. Med. Chem..

[152] Khan S., Ahmad K., Ahmad A., Raish M., Jan B.L., Khan A., Khan M.S. (2018). Biogenic pentagonal silver nanoparticles for safer and more effective antibacterial therapeutics. Int. J. Nanomed..

[153] Park D.H., Gautam M., Park S.J., Hwang J., Yong C.S., Kim J.O., Byeon J.H. (2019). Plug-and-play safe-by-design production of metal-doped tellurium nanoparticles with safer antimicrobial activities. Environ. Sci. Nano.

[154] Hadrup N., Sharma A.K., Loeschner K. (2018). Toxicity of silver ions, metallic silver, and silver nanoparticle materials after in vivo dermal and mucosal surface exposure: A review. Regul. Toxicol. Pharmacol..

[155] Singh S. (2019). Zinc oxide nanoparticles impacts: Cytotoxicity, genotoxicity, developmental toxicity, and neurotoxicity. Toxicol. Mech. Methods.

[156] Bai C., Tang M. (2020). Toxicological study of metal and metal oxide nanoparticles in zebrafish. J. Appl. Toxicol..

[157] Trickler W.J., Lantz S.M., Murdock R.C., Schrand A.M., Robinson B.L., Newport G.D., Schlager J.J., Oldenburg S.J., Paule M.G., Slikker W. (2010). Silver Nanoparticle Induced Blood-Brain Barrier Inflammation and Increased Permeability in Primary Rat Brain Microvessel Endothelial Cells. Toxicol. Sci..

[158] Crosera M., Bovenzi M., Maina G., Adami G., Zanette C., Florio C., Larese F.F. (2009). Nanoparticle dermal absorption and toxicity: A review of the literature. Int. Arch. Occup. Environ. Health.

[159] Niska K., Zielinska E., Radomski M.W., Inkielewicz-Stepniak I. (2018). Metal nanoparticles in dermatology and cosmetology: Interactions with human skin cells. Chem. Interact..

[160] Martin A., Sarkar A. (2017). Overview on biological implications of metal oxide nanoparticle exposure to human alveolar A549 cell line. Nanotoxicology.

[161] Schneider T., Westermann M., Glei M. (2017). In vitro uptake and toxicity studies of metal nanoparticles and metal oxide nanoparticles in human HT29 cells. Arch. Toxicol..

[162] You C., Liping Z., Wang X., Wu P., Ho J.K., Jin R., Zhang L., Shao H., Han C. (2017). Silver nanoparticle loaded collagen/chitosan scaffolds promote wound healing via regulating fibroblast migration and macrophage activation. Sci. Rep..

[163] Naraginti S., Kumari P.L., Das R.K., Sivakumar A., Patil S.H., Andhalkar V.V. (2016). Amelioration of excision wounds by topical application of green synthesized, formulated silver and gold nanoparticles in albino Wistar rats. Mater. Sci. Eng. C.

[164] Lee J., Kim J., Go J., Lee J.H., Han D.-W., Hwang D., Lee J. (2015). Transdermal treatment of the surgical and burned wound skin via phytochemical-capped gold nanoparticles. Colloids Surf. B Biointerfaces.

[165] Das S., Baker A.B. (2016). Biomaterials and Nanotherapeutics for Enhancing Skin Wound Healing. Front. Bioeng. Biotechnol..

[166] Ahamed M.N., Sankar S., Kashif P., Basha S., Sastry T. (2015). Evaluation of biomaterial containing regenerated cellulose and chitosan incorporated with silver nanoparticles. Int. J. Biol. Macromol..

[167] Raguvaran R., Manuja B.K., Chopra M., Thakur R., Anand T., Kalia A., Manuja A. (2017). Sodium alginate and gum acacia hydrogels of ZnO nanoparticles show wound healing effect on fibroblast cells. Int. J. Biol. Macromol..

[168] Punjataewakupt A., Napavichayanun S., Aramwit P. (2019). The downside of antimicrobial agents for wound healing. Eur. J. Clin. Microbiol. Infect Dis..

